# Surgical treatment of facial blushing: Patient selection and operative technique (retrospective observational study)

**DOI:** 10.1097/MD.0000000000029808

**Published:** 2022-07-08

**Authors:** Jae Kil Park, Kwanyong Hyun, Mi Hyoung Moon, Jungsun Lee

**Affiliations:** a Department of Thoracic and Cardiovascular Surgery, Seoul St. Mary’s Hospital, Catholic University of Korea, Seoul, South Korea

**Keywords:** compensatory hyperhidrosis, diffuse sympathicotomy, endoscopic thoracic sympathicotomy, facial blushing

## Abstract

Surgeons are often reluctant to offer further intervention to patients with medically intractable facial blushing. This is mainly because of the relatively high failure rate of blushing resolution and a high incidence of compensatory hyperhidrosis. In this study, we sought to identify the type of blushing that would benefit from surgery and minimize compensatory hyperhidrosis by applying diffuse sympathicotomy (DS).

This study was a retrospective review of 62 patients who underwent R2 endoscopic thoracic sympathicotomy (ETS) and preemptive DS for facial blushing. Facial blushing was classified as autonomic-mediated blushing (thermoregulatory, emotional) and vasodilator-mediated blushing (constant) based on the history and precipitating factors for blushing. DS was performed at lower-thoracic levels in the form of limited DS (right R5/7/9/11, left R5/6/8/10) or extended DS (bilateral R5-11).

Resolution of blushing (described as “almost disappeared”) was achieved in 48% of patients with a median follow-up of 19.6 months. There was a significant difference in resolution among 3 types of blushing (emotional: 55%, thermoregulatory: 28%, constant: 15%, *P* = .03). Multivariate analysis confirmed thermoregulatory and constant type blushing as a potential independent predictor of blushing resolution. Even though there was no difference between the DS procedures with respect to compensatory hyperhidrosis, intolerable compensatory hyperhidrosis (Hyperhidrosis Disease Severity Scale = 4) occurred in only 11% of patients. DS redistributed sweating area, being predominantly on the chest and mid-back (89%), also seen on the abdomen-waist-groin-buttocks-thighs (63%). Overall, 77% of patients experienced satisfactory results.

Emotional blushing proved to be an established indication of ETS where good long-term results can be expected. Expansion of surgical indication to thermoregulatory or constant type blushing needs to be validated in future studies. Additionally, compensatory hyperhidrosis, another hurdle for ETS, can be minimized by preemptive DS, resulting in redistribution and decrease of sweating.

## 1. Introduction

A blush is the reflection of vasodilation of cutaneous blood vessels, elicited by a variety of autonomic or vasodilatory stimuli. The innervation of the cutaneous blood vessels of the face is mediated through the upper thoracic sympathetic chain.^[1]^ Therefore, interruption of this nerve supply could essentially control sympathetically mediated autonomic facial blushing. However, surgical treatment is usually reserved for patients who are severely affected and in whom anxiety disorders are ruled out.

The trends in surgical approach have evolved during the last decade at many institutions, with endoscopic thoracic sympathicotomy (ETS) being the preferred method for treating facial blushing.^[2–10]^ However, compensatory hyperhidrosis as a side effect is of greater concern, with a severity greater than primary hyperhidrosis,^[11]^ which makes surgeons reluctant to offer surgery as a treatment for patients with blushing. Hence, relief of facial blushing by surgical interruption and simultaneous minimizing of compensatory hyperhidrosis are issues that need further addressing. This investigation was conducted to present the feasibility and outcomes of our experience with upper thoracic (R2) sympathicotomy and preemptive diffuse lower-thoracic sympathicotomy for patients with blushing.

## 2. Methods

### 2.1. Patients and data collection

A total of 247 patients underwent ETS at Seoul St. Mary’s Hospital, South Korea, from January 2018 to June 2019 for hyperhidrosis or facial blushing. Among these patients, 65 underwent operation for facial blushing. The indication for the operation was facial blushing perceived as disabling by the patient for at least 5 years. Patients with constant facial redness irrespective of emotional stress were seen by a dermatologist to exclude primary dermatological disease. Sixty-five percent of emotional facial redness had sought psychologic intervention, including medication, and failed to achieve blushing relief in spite of supportive treatments. All hospital records were retrieved retrospectively and the following data were recorded: symptoms, duration of the surgical procedure, length of hospital stay, and postoperative complications. This study was approved by the Institutional Review Board of our hospital (KC19RESI0542) and written informed consent for the ETS was obtained from each patient.

Facial blushing was classified as either autonomic-mediated blushing (emotional, thermoregulatory) or vasodilator-mediated blushing (constant) based on the detailed patient history and precipitating factors for blushing. Follow-up of blushing and side effects was carried out on return visits or telephonically at 3- and 6-month intervals after the surgery. The data collected included improvement of blushing, postoperative complications, severity of compensatory hyperhidrosis, and patient satisfaction level postsurgery. The improvement of blushing was classified into 3 categories, namely “almost disappeared”, “decreased, but still remained”, or “no effect of the operation”. The severity of compensatory hyperhidrosis was classified according to the Hyperhidrosis Disease Severity Scale (HDSS).^[12]^ To assess the severity of compensatory hyperhidrosis, patients were asked whether they had to change clothes sometimes during the day because of excessive sweating. When compensatory hyperhidrosis existed, the severity and location were further assessed. The location was classified into 3 zones: upper zone including the chest and mid-back; middle zone including the abdomen, waist, groin, buttock, and thigh; and lower zone including the popliteus, calf, and sole.

Statistical analysis included cross-tabulation, and the chi-square test was used in the computing environment *R* (*R* Development Core Team, Austria, 2008). Multivariate logistic regression analysis was performed for potential factors that had a univariate *P* value of <.2 in the preliminary univariate analysis. All *P* values <.05 were considered to indicate statistically significant.

### 2.2. Operative techniques

**Figure 1.**
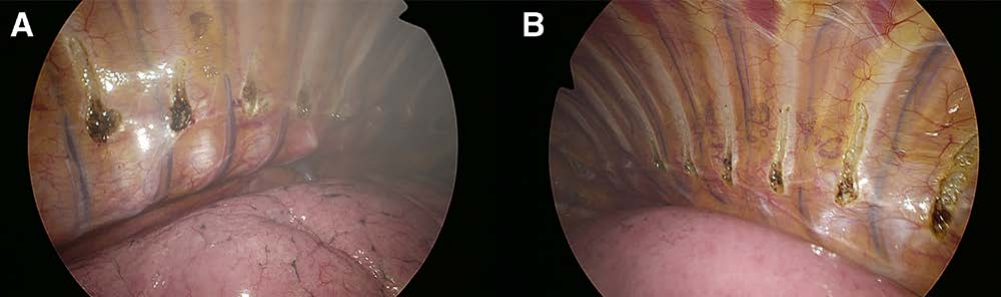
Thoracoscopic view of extended DS on the (A) right side and (B) left side. DS = diffuse sympathicotomy.

All patients were operated in the lateral decubitus position with abduction of the arm under general anesthesia with selective bronchial intubation using a double-lumen endotracheal tube. Starting on the left side, two 5-mm thoracoscopic ports were placed, and CO_2_ gas was insufflated into the thoracic cavity with <8 mm Hg of pressure to deflate the lung. The sympathetic chain was identified at the level of the crossing of the second costal head. The parietal pleura was opened, and the sympathetic chain was transected using monopolar electrocautery. The incision was extended laterally for approximately 2 cm on the second costa to include any accessory nerve fibers (the nerve of Kuntz). Then, diffuse sympathicotomy (DS) was performed at the lower-thoracic levels, including R5, R7, R9, and R11 on the right and R5, R6, R8, and R10 on the left (limited DS) as described in our previous report.^[13]^ The thoracic ganglia were disconnected by applying monopolar electrocoagulation on the sympathetic trunk at the level of the superior margin of each rib. During the study period, we modified our surgical technique after 9-month surveillance for procedure-related side effects. The levels of interruption were expanded to maximize the sympatholytic effect, by performing sympathicotomy on the levels of R5, R6, R7, R8, R9, R10, and R11 on both sides (extended DS) (Fig. 1). All procedures were completed by reinflation of the lung while the anesthesiologist ventilated the patient manually before a 12-French chest tube was subsequently removed. At times, pleural drain was left in place for 12 to 14 hours in patients with a persistent air leak from an injury to the visceral pleura. Most of the patients were admitted overnight and discharged the next morning if there were no side effects.

## 3. Results

During the 16-month study period, 65 patients (median age: 33 [18–61] years, 52% women and 48% men) were surgically treated for facial blushing at our institution. Of these, 62 were available for follow-up evaluation of the symptomatic outcome analysis. Results are given for 62 patients (Table 1). The types of blushing were emotional in 51 patients, thermoregulatory in 36 patients, and constant blushing in 20 patients. There was no significant correlation among the 3 types of blushing (Person product-momentum correlation: emotional vs thermoregulatory, *R* = –0.22; emotional vs constant, *R* = –0.22; thermoregulatory vs constant, *R* = 0.24). Further, 48 patients (77%) had isolated blushing and the remaining 14 patients (23%) had concomitant primary hyperhidrosis in the craniofacial, palmar, or plantar areas.

**Table 1:** Patient characteristics and perioperative details.

Characteristic	Results
Median age, years (range)	33 (18–61)
Gender, M/F	30/32
Etiology of facial blushing, n (%)	
Thermoregulatory	36 (58%)
Emotional	51 (82%)
Constant	20 (32%)
Isolated* facial blushing, n (%)	48 (77%)
Upper thoracic sympathicotomy, n (%)	
R2 (on the second rib)	62 (100%)
Diffuse sympathicotomy on lower-thoracic levels, n (%)	
Limited DS (right R5/7/9/11, left R5/6/8/10)	41 (66%)
Extended DS (both sides R5-11)	21 (34%)
Median operation time,† min (range)	45 (32–280)
Placement of pleural drainage, n (%)	4 (6%)
Median hospital stay, days (range)	1 (0–3)

DS, diffuse sympathicotomy, F = female, M = male.

* Without combined primary hyperhidrosis.

† Measured as bilateral procedure time.

Sympathicotomy for facial blushing was performed at R2 in all patients in the study group. At the same time, DS on the lower-thoracic levels was performed to prevent compensatory hyperhidrosis: limited DS in 41 patients and extended DS in 21 patients. The average operation time was 50 minutes, excluding anesthesia induction and reversal time. At the end of the procedure, 4 patients (6%) were fitted with Jackson-Pratt drainage to prevent postoperative pneumothorax. The majority of patients were discharged on the first postoperative day (range: 0–3 days). Transient sinus bradycardia after an uneventful R2 sympathicotomy developed in 2 patients, in which bradycardia was managed with transient inotropic medications to prevent hypotension. In these 2 patients, alpha-receptor agonist (midodrine) was taken for 1 week postoperatively and discontinued after sinus rhythm was confirmed at the outpatient clinic visit. Other adverse events included muscle weakness (n = 1), bloating (n = 1), and delayed ejaculation (n = 1), all of which eventually resolved after several months of follow-up.

Sixty-two patients answered the questionnaire administered by a telephonic or outpatient survey. The median interval between sympathetic intervention and questionnaire-based survey was 19.6 (range: 7.5–23.3) months. Overall outcomes after surgery are summarized in Table 2. Briefly, 85% of patients showed marked improvement in blushing (i.e., either disappeared or decreased). However, in a subgroup analysis based on blushing type, as shown in Table 3, there was a significant difference among the 3 types of blushing (emotional vs thermoregulatory vs constant) with regard to postoperative improvement (Pearson chi-square = 23.6, 4 degree of freedom; *P* < .001). Since some patients belonged to multiple types of blushing, subpopulation analysis was conducted and revealed that patients with sole emotional blushing showed superior postoperative blushing resolution. In univariate and multivariate logistic regression analysis for blushing resolution (as “almost disappeared” on reported effects), 2 types of blushing (thermoregulatory blushing and constant blushing) had a negative impact on blushing resolution independently. Age at surgery, gender, body mass index and the presence of combined hyperhidrosis had no significant impact on blushing resolution (Table 4).

**Table 2:** Postoperative follow-up.

Variable	Value
Median follow-up, mo (range)	19.6 (7.5–23.3)
Improvement of blushing	
Almost disappeared	30 (48%)
Decreased, but still remained	23 (37%)
No effect of the operation	9 (15%)
Recurrence of facial blushing	9 (15%)
Occurrence of compensatory hyperhidrosis	
HDSS* 1–2	33 (53%)
HDSS 3	22 (36%)
HDSS 4	7 (11%)
Overall satisfaction levels	
Very satisfied	8 (13%)
Satisfied	40 (64%)
Dissatisfied	14 (23%)

Data are presented as n (%) except where otherwise noted.

HDSS = Hyperhidrosis Disease Severity Scale.

* HDSS 1 = my sweating is never noticeable and never interferes with my daily activities, HDSS 2 = my sweating is tolerable but sometimes interferes with my daily activities, HDSS 3 = my sweating is barely tolerable and frequently interferes with my daily activities, HDSS 4 = my sweating is intolerable and always interferes with my daily activities.

**Table 3:** Resolution of facial blushing based on blushing type.

Improvement of blushing	Blushing type			*P* value				
	E (n = 51)	T (n = 36)	C (n = 20)					
Almost disappeared	28 (55%)	10 (28%)	3 (15%)	<.001				
Decreased, but still remained	18 (35%)	20 (55%)	11 (55%)					
No effect of the operation	5 (10%)	6 (17%)	6 (30%)					
	E only (n = 21)	T only (n = 5)	C only (n = 2)	E + T (n = 16)	E + C (n = 3)	T + C (n = 4)	E + T +C (n = 11)	*P* value
Almost disappeared	18 (86%)	1 (20%)	1 (50%)	8 (50%)	1 (33%)	0	1 (9%)	
Decreased, but still remained	2 (9%)	2 (40%)	0	8 (50%)	1 (33%)	3 (75%)	7 (64%)	<.001
No effect of the operation	1 (5%)	2 (40%)	1 (50%)	0	1 (33%)	1 (25%)	3 (27%)	

Data are presented as n (%) except where otherwise noted.

C = constant, E = emotional, T = thermoregulator.

**Table 4:** Factors affecting blushing resolution (almost disappeared).

	Univariate logistic regression			Multiple logistic regression		
Variable	Estimate	*P* value	Odds ratio	Estimate	*P* value	Odds ratio
Age	–0.02	0.43	0.98			
Gender	0.38	0.45	1.47			
BMI	0.01	0.93	1.01			
Isolated blushing (vs combined blushing)	0.29	0.64	1.33			
Extended DS* (vs limited DS†)	0.53	0.33	1.7			
Emotional blushing	1.7	0.04	5.48	0.87	0.34	2.38
Thermoregulatory blushing	–2.16	<.001	0.12	–2.01	0.003	0.13
Constant blushing	–2.32	<.001	0.1	–2.13	0.007	0.12

BMI = body mass index, DS, diffuse sympathicotomy.

* Interruption level is right R5/7/9/11, left R5/6/8/10 levels.

† Interruption level is both sides R5–11 levels.

**Figure 2.**
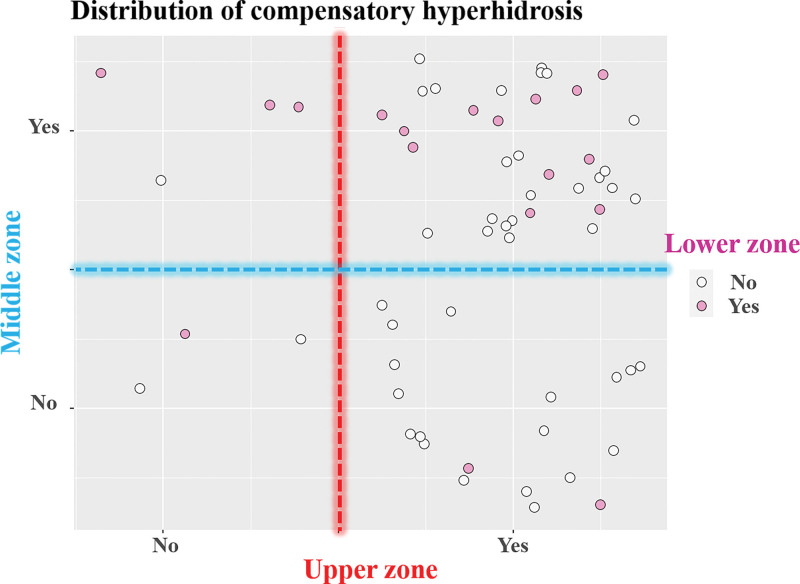
The distribution of compensatory hyperhidrosis. Upper zone includes chest and mid-back; middle zone includes abdomen, waist, groin, buttock, and thigh; and lower zone includes popliteus, calf, and sole.

Recurrence of blushing occurred in 9 patients (15%) within the first year. Compensatory hyperhidrosis that interferes with daily activities (HDSS 3 and 4) occurred in 47% of responding patients; incidence of HDSS 3 and HDSS 4 were 36% and 11%, respectively (Table 2). Table 5 shows differences in the occurrence of compensatory hyperhidrosis according to the patient factors. Neither age at surgery, sex, nor isolated blushing correlated significantly with the degree of compensatory hyperhidrosis. There was also no significant difference in preventing compensatory hyperhidrosis between the 2 extents of DS (*P* = .68). The distribution of compensatory hyperhidrosis is presented in Figure 2. Compensatory hyperhidrosis predominantly occurred on the chest and mid-back (upper zone, 89%) but was also seen on the abdomen, waist, groin, buttocks, and thighs (middle zone, 63%). The incidence of compensatory hyperhidrosis on the popliteus, calf, and sole (lower zone, 29%) was low but not negligible. Gustatory sweating occurred in 23% of patients but was not related to the severity of compensatory hyperhidrosis (*P* = .16). Overall, 77% of patients reported satisfactory results with sympathetic surgery for facial blushing and preemptive DS.

**Table 5:** Differences in the occurrence of compensatory hyperhidrosis.

		Degree of compensatory hyperhidrosis			
Variables	Overall (n = 62)	HDSS* 1–2 (n = 33)	HDSS 3 (n = 22)	HDSS 4 (n = 7)	*P* value
Operation time, min	49.8 ± 33.9	50.1 ± 42.3	50.2 ± 23.9	47.1 ± 10.4	.977
Isolated blushing, n (%)	48 (77%)	27 (82%)	18 (82%)	3 (43%)	.067
Type of DS, n (%)					
Limited DS†	41 (66%)	23 (70%)	13 (59%)	5 (71%)	.683
Extended DS‡	21 (34%)	10 (30%)	9 (41%)	2 (29%)	
Gustatory sweating, n (%)	14 (23%)	5 (15%)	8 (36%)	1 (14%)	.157
Overall satisfaction, n (%)					
Very satisfied	8 (13%)	6 (18%)	2 (9%)	0	.001
Satisfied	40 (64%)	22 (67%)	17 (77%)	1 (14%)	
Dissatisfied	14 (23%)	5 (15%)	3 (14%)	6 (86%)	

DS = diffuse sympathicotomy, HDSS = Hyperhidrosis Disease Severity Scale.

* HDSS 1 = my sweating is never noticeable and never interferes with my daily activities, HDSS 2 = my sweating is tolerable but sometimes interferes with my daily activities, HDSS 3 = my sweating is barely tolerable and frequently interferes with my daily activities, HDSS 4 = my sweating is intolerable and always interferes with my daily activities.

† Interruption level is right R5/7/9/11, left R5/6/8/10 levels.

‡ Interruption level is both sides R5–11 levels.

## 4. Discussion

This study involved a cohort of patients with facial blushing treated with conventional R2 sympathicotomy and preemptive diffuse, lower-thoracic sympathicotomy. The rarity of facial blushing and the related data on compensatory hyperhidrosis development make our findings valuable. This study showed that DS was effective in minimizing compensatory hyperhidrosis development and that etiologic factors which precipitate facial blushing significantly affect the postoperative time course of blushing in terms of resolution.

While the beneficial effect of ETS on palmar hyperhidrosis has been documented in many previous studies, its effect on facial blushing has been less rewarding. First, results from the literature indicate that ETS works poorly on slowly emerging and long-lasting facial blushing as well as on upper chest and neck blushing, showing a 14.0% primary failure.^[14]^ Second, recurrence of facial blushing is frequent, reportedly 30% within the first year.^[9]^ Last, the incidence of developing compensatory hyperhidrosis is high, and the overall satisfaction rates are lower than expected. Previous reports have revealed that approximately 10% to 15% of the patients regretted the operation.^[7,11]^ Considering these, the side effects of ETS are of greater concern than the beneficial effects on facial blushing in some patients.

Facial blushing is a consequence of increased cutaneous blood flow secondary to vasodilation and represents part of a synchronized physiologic response of cutaneous vascular smooth muscle to a variety of autonomic or vasodilator stimuli. As the indications of ETS for facial blushing have not yet been fully clarified, we assessed the outcome of ETS by the etiology of facial blushing. In our study, patients with emotional blushing were more likely to achieve postoperative resolution than those who presented with thermoregulatory or constant blushing. Drott et al^[3]^ also described blushing resolution in detail based on different situations, which led to sympathetic stimuli. The various situations were “socially tense situations,” “physical exercise,” “temperature changes,” and “alcohol intake.” Although the resolution of blushing was significant in all these situations, the degree of resolution measured by visual analogue scale score was different. In our analysis of blushing resolution, we confirmed etiology of blushing as an important predictor for resolution. Briefly, 55%, 28%, and 15% of patients respectively in the emotional, thermoregulatory, and constant blushing subgroups experienced the “almost disappeared” outcome after ETS. And in subgroup analysis, patients with sole emotional blushing showed extremely high rate of blushing resolution (86%, “almost disappeared”) compared to sole thermoregulatory or sole constant blushing (20% and 50%, respectively). This highlights that autonomic-mediated blushing can be better controlled with surgical interruptions, and surgical management of blushing in selected patient groups can result in improved outcomes.

The pathophysiology behind compensatory hyperhidrosis is poorly understood. However, local hyperhidrosis is thought to be caused by increased sympathetic signaling to the sweat glands. Interestingly, the sites affected by compensatory hyperhidrosis before ETS are generally the thermoregulatory, nonglabrous skin regions of the trunk/back, buttocks, groin, and thighs that sweat normally. A plausible explanation is that normal thermoregulatory effectors become upregulated as a response to the anhidrosis caused by ETS.^[15]^ Hence, we tried to eliminate these reflex sweating responses by controlling segmental origins of the sympathetic nervous system; however, the sympathetic dermatomes overlap with each other and are difficult to systematize. Sympathetic supply to the head and neck has its origin from the nerve roots of T1–T5, the upper limb from roots T3-T6, the thorax from roots T3–T6, the abdomen from roots T7–T11, and the lower limbs from roots T10–L2 or L3. Based on this information, we started to perform limited DS to minimize compensatory hyperhidrosis, which involved multiple bilateral sympathicotomy at the levels of R5, R7, R9, and R11 (all right) and R5, R6, R8, and R10 (all left). In an attempt to prevent the side effects of DS, not all the levels on the right and left sides were surgically interrupted. With time and establishment of the safety of limited DS, we modified our mode of surgical intervention to interrupt all levels of sympathetic segments, in the form of extended DS, which theoretically allows for complete sympathetic denervation of the trunk and abdomen. In our patients, the side effects after DS were rare, but potential dangers such as the occurrence of postoperative pain or gastrointestinal disturbance should not be underestimated.

The present study showed that the phenomenon of compensatory hyperhidrosis in R2 resection also occurs after DS but causes fewer patients to be in severe distress. The reported incidence of compensatory hyperhidrosis was extremely high with R2 and/or R3 resection in previous investigations. Licht et al^[7]^ reported that 88% of the study patients had compensatory hyperhidrosis and it was so severe in 25% that they often had to change clothes during the day. Licht et al^[9]^ again reported the incidence of compensatory hyperhidrosis was as high as 95% in their prospective study. Comparing these findings to ours, 47% of patients reported interference with daily activities and only 11% were found to have intolerable sweating necessitating a change of clothes. In our experience, DS has a positive impact on reducing the severity of compensatory hyperhidrosis (judged by HDSS) and the affected area of compensatory hyperhidrosis appears to be moved from the upper zone to the middle zone. Together with the results of our previous report,^[13]^ this study again proposes clinical safety of DS with minor self-limiting complications (muscle weakness, bloating, and delayed ejaculation).

It must be emphasized that the present study is a presentation of the impact of blushing subtype on operative outcomes, but, at the same time, this is an initial presentation of a newly introduced surgical modality for preventing compensatory hyperhidrosis in patients with facial blushing. Therefore, this study has several shortcomings. The sample size was relatively small, and there was no randomization. In addition, for consistency, outcomes should be assessed by obtaining objective proof of sympathetic denervation and diminished blushing. For a further and more reliable exploration of the subject, a study in a larger population with objective outcome measurement is required, with longer follow-up, to determine the long-term operative effects of sympathetic interventions.

## 5. Conclusion

Emotional blushing is an established indication of ETS where good long-term results can be expected. Thermoregulatory and constant blushing are likely questionable indications for surgery. A larger series of patients with facial blushing is required before ETS can be recommended in this subset of patients. Furthermore, we present encouraging evidence of a compensatory hyperhidrosis improvement after preemptive diffuse lower-thoracic sympathicotomy in patients with facial blushing. However, further studies in a larger sample of patients and longer follow-up investigations are necessary to confirm the effect and safety of DS.

### Author contributions

Conceptualization: Jae Kil Park, Kwanyong Hyun.

Data curation: Kwanyong Hyun, Jungsun Lee.

Formal analysis: Mi Hyoung Moon.

Investigation: Kwanyong Hyun, Mi Hyoung Moon.

Methodology: Jae Kil Park, Jungsun Lee.

Project administration: Jungsun Lee, Mi Hyoung Moon.

Resources: Jae Kil Park, Kwanyong Hyun, Mi Hyoung Moon.

Software: Kwanyong Hyun.

Supervision: Jae Kil Park, Mi Hyoung Moon.

Validation: Mi Hyoung Moon, Jae Kil Park.

Visualization: Kwanyong Hyun.

Writing – original draft: Kwanyong Hyun, Jungsun Lee.

Writing – review & editing: Kwanyong Hyun, Jae Kil Park, Mi Hyoung Moon.
